# Comparative efficacy and safety of first, second, and third-generation EGFR tyrosine kinase inhibitors in treatment-naïve EGFR-mutant advanced non–small cell lung cancer: a systematic review and network meta-analysis of randomized controlled trials

**DOI:** 10.1186/s43046-026-00414-2

**Published:** 2026-09-14

**Authors:** Sumit Mahato, Shiv Mudgal, Harminder Singh, Seshadri Reddy Varikasuvu, Hansraj Kumar, Jameema Thankachan, Subodh Kumar

**Affiliations:** 1 Deaprtment of Pharmacology, All India Institute of Medical Sciences, Deoghar, India; 2 College of Nursing, Dr. Ram Manohar Lohia Institute of Medical Sciences, Lucknow, India; 3 Department of Biochemistry, and Department of Evidence Synthesis (DOES), DHR-TRC, DHR-HTAIn, All India Institute of Medical Sciences, Deoghar, India; 4 Department of Pharmacology, and Department of Evidence Synthesis (DOES), DHR-TRC, DHR-HTAIn, All India Institute of Medical Sciences, Deoghar, India

**Keywords:** EGFR, NSCLC, Network meta-analysis, TKIs

## Abstract

**Background:**

The optimal first-line epidermal growth factor receptor (EGFR) tyrosine kinase inhibitor (TKI) for EGFR-mutant advanced non–small cell lung cancer (NSCLC) remains uncertain due to the availability of multiple generations of TKIs and limited head-to-head comparisons.

**Methods:**

A systematic review and network meta-analysis (NMA) of randomized controlled trials (RCTs) was conducted comparing first, second, and third-generation EGFR-TKIs in treatment-naïve EGFR-mutant NSCLC. Databases including PubMed, Embase, Scopus, and the Cochrane Central Register of Controlled Trials were searched. Outcomes included overall survival (OS), progression-free survival (PFS), and grade ≥ 3 adverse events (AEs). Hazard ratios (HRs) and odds ratios (ORs) were pooled using a frequentist random-effects NMA. Treatments were ranked using P-scores.

**Results:**

Nine RCTs were included for PFS and eight for OS and AEs. For OS, dacomitinib (HR 0.75, 95% CI 0.59–0.95) and osimertinib (HR 0.80, 95% CI 0.67–0.96) showed significant benefit versus first-generation TKIs. For PFS, all evaluated second- and third-generation TKIs significantly improved PFS, with furmonertinib (HR 0.44, 95% CI 0.34–0.57) having the highest P-score. In safety analysis, no EGFR-TKI showed a statistically significant difference in grade ≥ 3 adverse events compared with first-generation TKIs. Although befotertinib and dacomitinib had numerically higher odds, all confidence intervals were wide and crossed the null value.

**Conclusions:**

While several EGFR-TKIs improved efficacy over first-generation agents, no single treatment was clearly superior across all efficacy and safety outcomes. Osimertinib showed consistent efficacy, while its safety advantage became evident in the sensitivity analysis excluding FLAURA China.

**Supplementary Information:**

The online version contains supplementary material available at https://doi.org/10.1186/s43046-026-00414-2.

## Introduction

Lung cancer is one of the leading causes of cancer-related death worldwide, accounting for 2.5 million new incidences with 1.8 million deaths in 2022 which is more than deaths due to breast, prostate, and colorectal cancers combined [1]. Non–small-cell lung cancer (NSCLC) constitutes approximately 80 to 85% of lung cancers, with adenocarcinoma as the predominant histologic subtype [1, 2]. In patients with advanced NSCLC, EGFR-activating mutations particularly exon 19 mutation and exon 21 substitutions, are among the most common targetable oncogenic alterations. These mutations show wide geographic variation, occurring in about 40 to 50% of tumours in East Asian populations and 10 to 20% of tumours in Western populations [3–5]. Introduction of tyrosine kinase inhibitors (TKIs) for EGFR-mutated advanced NSCLC completely changed the outlook for its treatment. First-generation EGFR tyrosine kinase inhibitors (TKIs), such as gefitinib and erlotinib, improved its overall survival and progression-free survival when compared to platinum-based chemotherapy in patients with EGFR-mutated advanced NSCLC. It established EGFR-TKI monotherapy as the standard first-line treatment (e.g., IPASS, EURTAC) [6, 7]. However, acquired resistance and limited central nervous system activity constrained the benefit with these agents [7].

Second-generation irreversible EGFR TKIs, including afatinib and dacomitinib were later developed to provide broader ErbB-family inhibition and increased potency. Randomized trials showed longer progression-free and overall survival compared with chemotherapy or first-generation TKIs. However, this clinical benefit came with a trade-off, patients experienced higher rates of severe (grade 3 or 4) diarrhea and rash. (LUX-Lung 3 and 6, ARCHER 1050) [8–11]. Third-generation TKIs, most notably osimertinib, and more recently developed agents such as lazertinib, aumolertinib, and furmonertinib, were designed to target both sensitizing EGFR mutations and the T790M resistance mutation while sparing wild-type EGFR. In FLAURA trial first-line osimertinib significantly improved progression-free and overall survival and reduced the risk of central nervous system progression compared with first-generation TKIs [12, 13]. Multiple EGFR TKIs across three generations, including newer third-generation agents such as lazertinib, aumolertinib, and furmonertinib are also available for first-line treatment of EGFR-mutated advanced NSCLC, but osimertinib is widely recommended preferred option in international guidelines [13, 14].

Despite these advances, important differences remain among available EGFR TKIs with respect to its efficacy for different outcomes, toxicity profiles, and accessibility [10–14]. Some conventional and network meta-analyses have attempted to compare EGFR TKI strategies, but they have either broad i.e. included chemotherapy and placebo as treatment arms or do not fully incorporate newer agents and regimens [15–17]. In this context, a focussed network meta-analysis which integrates both direct and indirect evidence from randomized controlled trials to determine relative efficacy and safety profiles of first, second, and third generation EGFR tyrosine kinase inhibitors (TKIs) in treatment-naïve patients with EGFR-mutated advanced non-small cell lung cancer (NSCLC) was required.

Accordingly, we performed a systematic review and network meta-analysis of randomized controlled trials to exclusively compare progression-free survival, overall survival and adverse events across these three generations of EGFR TKIs. The primary goal was to identify which treatment offers the most favourable balance between effective disease control and acceptable tolerability.

## Methods

### Search strategy and selection criteria

This systematic review and network meta-analysis was done in accordance with the PRISMA extension statement for network meta-analysis. A comprehensive literature search was performed in PubMed, Embase, Scopus, and the Cochrane Central Register of Controlled Trials (CENTRAL) from inception to 1 March 2026, with language restrictions (English only) (Supplementary Appendix 1). The search strategy combined Medical Subject Headings and free-text terms related to non–small cell lung cancer, EGFR mutations, and individual EGFR tyrosine kinase inhibitors (afatinib, dacomitinib, osimertinib, gefitinib, erlotinib, aumolertinib, furmonertinib, icotinib, and befotertinib) using Boolean operators. Reference lists of relevant studies were also screened to identify any eligible trials. We included randomized controlled trials enrolling adult patients with treatment naïve EGFR-mutant advanced or metastatic non–small cell lung cancer that compared EGFR tyrosine kinase inhibitors across generations and reported at least one outcome of interest. The primary outcome was overall survival, and secondary outcomes were progression-free survival and grade ≥ 3 adverse events, as defined in the individual trials. Studies that were non-randomized, single-arm, or non TKI controlled were excluded. The protocol was registered in PROSPERO (CRD42024553180). 

### Study selection and quality assessment

Two reviewers independently screened titles and abstracts and assessed full texts for eligibility, and disagreements were resolved through discussion with a third reviewer. Risk of bias was evaluated using the Cochrane Risk of Bias 2 tool. 

### Data extraction and synthesis

Data extraction was performed independently by two reviewers using a predefined data extraction form and cross-verified for accuracy. Extracted data included study characteristics, patient demographics, treatment arms, and reported effect estimates. Hazard ratios for overall and progression-free survival and odds ratios for adverse events were extracted, and log-transformed effect sizes with corresponding standard errors were calculated when required. For the safety analysis, the number of participants experiencing at least one grade ≥ 3 adverse event was extracted. The adverse-event definition used in each trial, including treatment-related, treatment-emergent, or all-cause events, was recorded because these definitions were not fully uniform across studies. 

### Assessment of transitivity

The transitivity assumption was assessed by comparing clinically important effect modifiers across treatment comparisons, including EGFR mutation subtype, smoking status, sex, geographic region, trial design, baseline central nervous system metastases, and maturity of survival data. The distribution of these characteristics was considered sufficiently comparable to support indirect comparisons, although incomplete reporting of some variables was acknowledged.

### Data analysis

All analyses were conducted using R (version 4.2.1). A frequentist random-effects network meta-analysis was performed using the ‘netmeta’ package to estimate pooled treatment effects. Hazard ratios were used for time-to-event outcomes and odds ratios for adverse events. Treatments were ranked using P-scores. Between-study heterogeneity was assessed using the I² statistic and τ². Consistency between direct and indirect evidence was evaluated using the design-by-treatment interaction model. Publication bias was assessed using comparison-adjusted funnel plots. Certainty of evidence was assessed using GRADE based framework adapted for network meta-analysis. Gefitinib, erlotinib, and icotinib were grouped into a common first-generation EGFR-TKI node because of their shared reversible mechanism of EGFR inhibition and similar clinical positioning. A fully separated-node analysis was not feasible because FLAURA reported a combined gefitinib/erlotinib comparator and icotinib was represented only in the befotertinib trial. A sensitivity analysis excluding the befotertinib-versus-icotinib trial was therefore performed to assess whether inclusion of icotinib materially influenced the network estimates. Post hoc sensitivity analyses excluding FLAURA China were performed for OS, PFS, and grade ≥ 3 adverse events because of partial participant overlap with the global FLAURA trial.

## Results

### Study selection

**Fig. 1:**
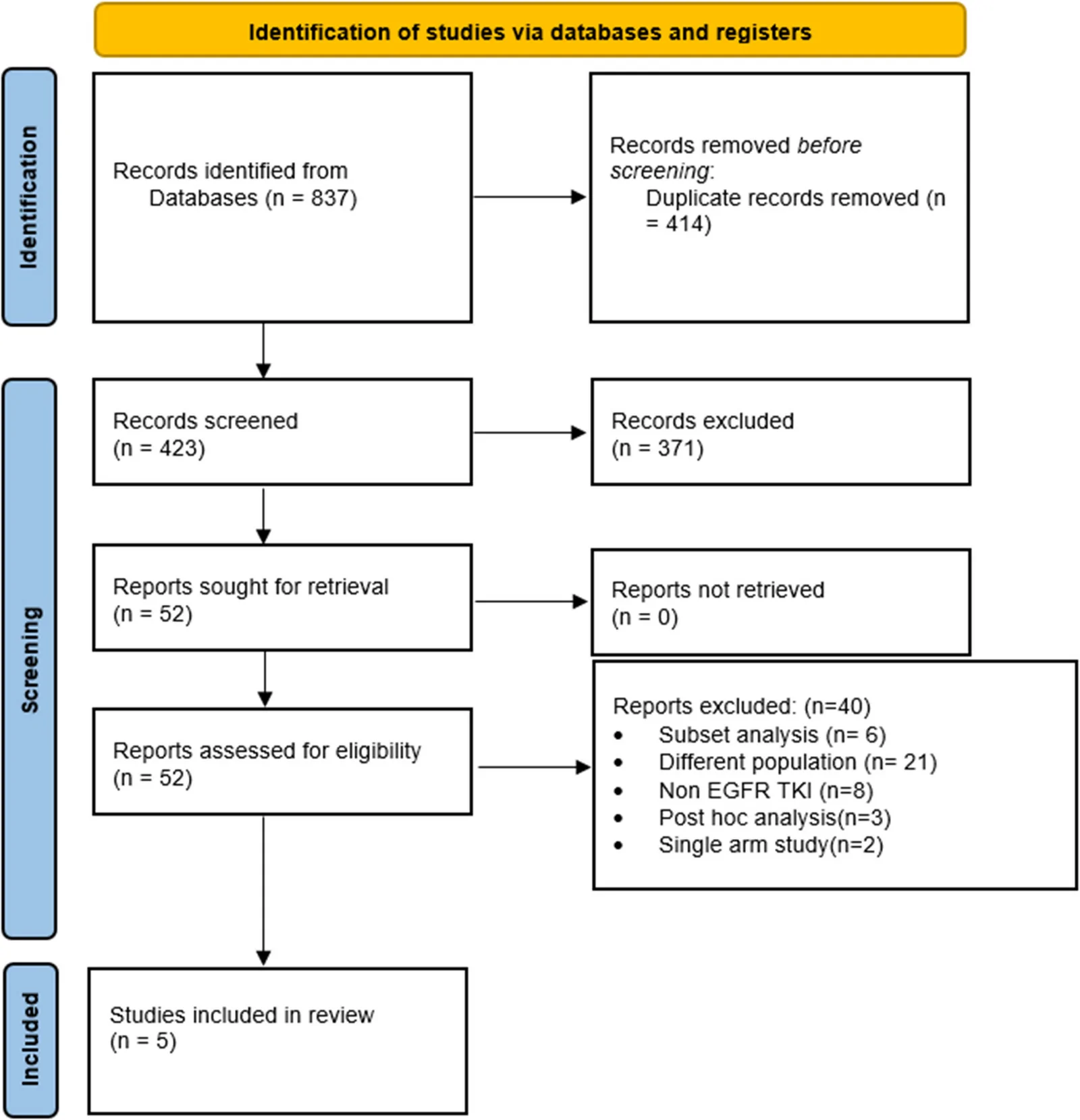
PRISMA flow diagram

Randomized controlled trials comparing EGFR-TKIs in treatment-naïve EGFR-mutant advanced NSCLC were included. A total of 837 records were identified through database searching. After removal of duplicates, 423 records were screened for title and abstract, and 52 full-text articles were assessed for eligibility. Twelve publications corresponding to nine randomized trial reports were included. FLAURA China included 136 participants, of whom 19 were also included in the global FLAURA trial and 117 were additionally recruited under the same protocol. Both reports were retained in the primary analysis because the regional report contributed 117 additional participants. However, the 19 overlapping participants introduced limited non-independence, and sensitivity analyses excluding FLAURA China were therefore performed. (Fig. 1).

### Study characteristics

Included trials were mostly phase II & III RCTs involving treatment-naïve EGFR-mutant NSCLC patients (stage IIIB–IV). All studies included exon 19 deletions and L858R mutations (Exon 21 substitution). Although the primary MARIPOSA study evaluated amivantamab plus lazertinib versus osimertinib, the present analysis included only the published exploratory comparison between the separately randomized lazertinib and osimertinib monotherapy arms. Study characteristics are summarized in Table 1.

**Table 1:** Characteristics of included randomized controlled trials

S. No.	First author	NCT ID	Population / Treatment / Stage	Mutation (%)	Gender (%)	Smoking (%)	Design	Intervention	Control	*n* (Intervention)	**n (Control)**
1	(a) Soria JC et al. 2018 [12]	NCT02296125	EGFR-mutant NSCLC; treatment-naïve; stage IIIB–IV	Ex19del ~ 63%;	Male ~ 37%;	Smoker ~ 36%;	Phase III, DB RCT	Osimertinib 80 mg OD	Gefitinib/Erlotinib	279	277
	(b) Ramalingam et al. 2020 [13](FLAURA)			L858R ~ 37%	Female ~ 63%	Non-smoker ~ 64%					
2	Cheng Y et al. 2021 [18] (FLAURA China)	NCT02296125	EGFR-mutant NSCLC; treatment-naïve; stage IIIB–IV	Ex19del ~ 51%;	Male ~ 35%;	Smoker ~ 25%; Non-smoker ~ 75%	Phase III, DB RCT	Osimertinib 80 mg OD	Gefitinib/Erlotinib	71	65
				L858R ~ 49%	Female ~ 65%						
3	a. Mok TS et al. 2017 [11]	NCT01774721	EGFR-mutant NSCLC; treatment-naïve; stage IIIB–IV	Ex19del ~ 59%;	Male ~ 40%;	Smoker ~ 35%;	Phase III, OL RCT	Dacomitinib 45 mg OD	Gefitinib 250 mg	227	225
	b. Wu YL et al. 2017 [19] (ARCHER 1050)			L858R ~ 41%	Female ~ 60%	Non-smoker ~ 65%					
4	a. Park K et al. 2016 [10]	NCT01466660	EGFR-mutant NSCLC; treatment-naïve; stage IIIB–IV	Ex19del ~ 58%;	Male ~ 40%;	Smoker ~ 35%;	Phase IIb, OL RCT	Afatinib 40 mg OD	Gefitinib 250 mg	160	159
	b. Paz-Ares L et al. 2017 [20] (LUX-Lung 7)			L858R ~ 42%	Female ~ 60%	Non-smoker ~ 65%					
5	Lu S et al. 2022 [21] (AENEAS)	NCT03849768	EGFR-mutant NSCLC; treatment-naïve; stage IIIB–IV	Ex19del ~ 65%;	Male ~ 37%;	Smoker ~ 30%;	Phase III, DB RCT	Aumolertinib 110 mg OD	Gefitinib 250 mg	214	215
				L858R ~ 35%	Female ~ 63%	Non-smoker ~ 70%					
6	Cho BC et al. 2023 [22] (LASER301)	NCT04248829	EGFR-mutant NSCLC; treatment-naïve; stage IIIB–IV	Ex19del ~ 62%;	Male ~ 35%;	Smoker ~ 30%;	Phase III, DB RCT	Lazertinib 240 mg OD	Gefitinib 250 mg	196	197
				L858R ~ 38%	Female ~ 65%	Non-smoker ~ 70%					
7	Lee SH et al. 2025 [23] (MARIPOSA)	NCT04487080	EGFR-mutant NSCLC; treatment-naïve; stage IIIB–IV	Ex19del ~ 60%;	Male ~ 38%;	Smoker ~ 32%;	DB RCT (exploratory)	Lazertinib 240 mg OD	Osimertinib 80 mg OD	216	429
				L858R ~ 40%	Female ~ 62%	Non-smoker ~ 68%					
8	Lu S et al. 2023 [24] (Befotertinib)	NCT04206072	EGFR-mutant NSCLC; treatment-naïve; stage IIIB–IV	Ex19del 64%;	Male ~ 40%;	Smoker ~ 33%;	Phase III, OL RCT	Befotertinib 75–100 mg OD	Icotinib 125 mg TDS	182	180
				L858R 36%	Female ~ 60%	Non-smoker ~ 67%					
9	Shi Y et al. 2022 [25] (FURLONG)	NCT03787992	EGFR-mutant NSCLC; treatment-naïve; stage IIIB–IV	Ex19del ~ 51%;	Male ~ 35%;	Smoker ~ 25%;	Phase III, DB RCT	Furmonertinib 80 mg OD	Gefitinib 250 mg	178	179
				L858R ~ 49%	Female ~ 65%	Non-smoker ~ 75%					

Trials with multiple publications (FLAURA, LUX-Lung 7, ARCHER 1050) were consolidated at the study level

*NSCLC *non–small cell lung cancer, *EGFR *epidermal growth factor receptor, *RCT *randomized controlled trial, *DB *double-blind, *OL *open-label, *OD *once daily, *TDS *three times daily

### Network geometry

**Fig. 2:**
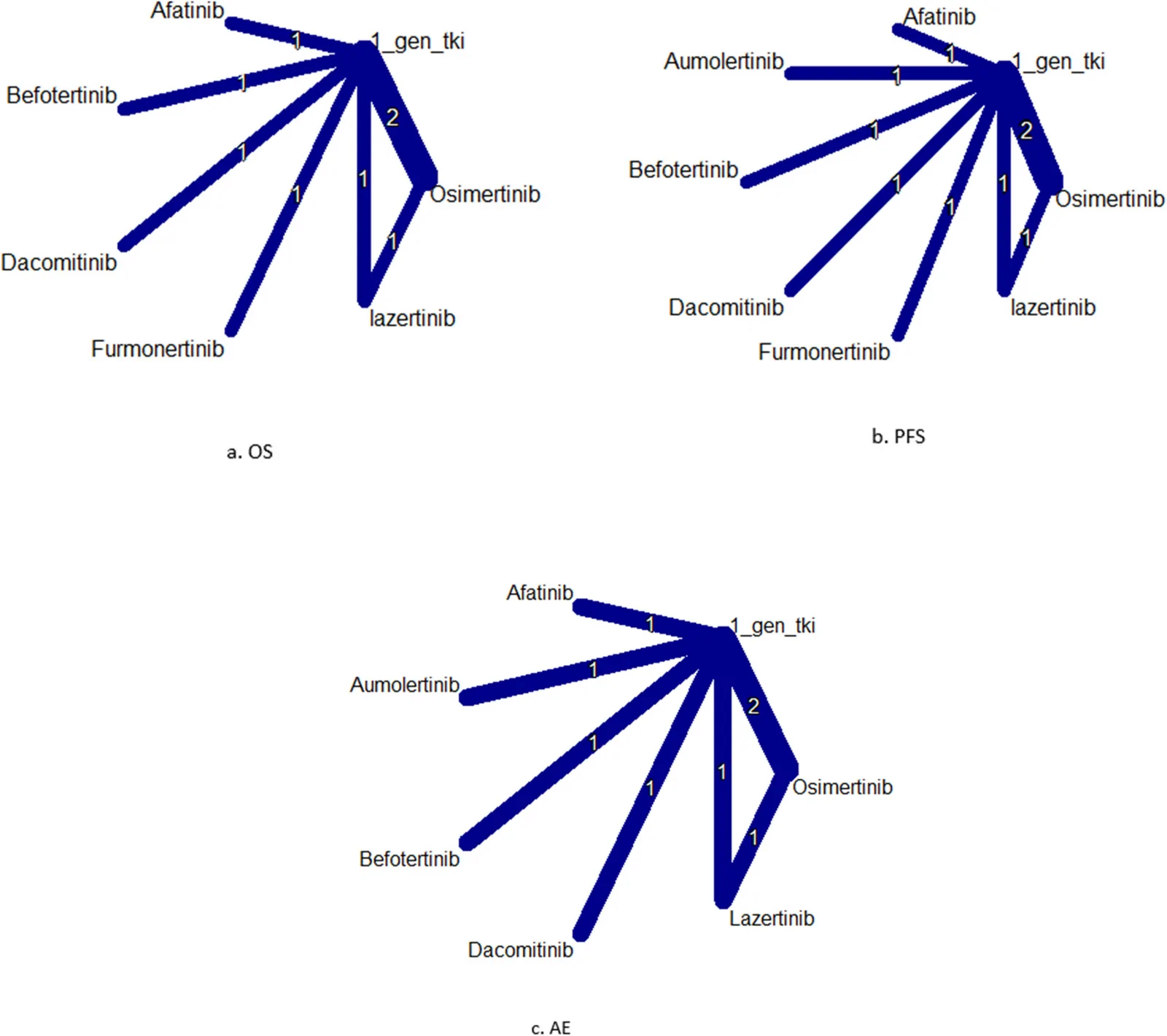
Network plots of treatment comparisons for overall survival (OS), progression-free survival (PFS), and grade ≥ 3 adverse events (AE). Nodes represent treatments, and edges represent direct comparisons between treatments. The thickness of the edges is proportional to the number of studies contributing to each comparison. The numbers on the edges indicate the number of studies

The networks were connected but sparse, with first-generation TKIs serving as the principal common comparator. Only one direct comparison between newer-generation agents, lazertinib versus osimertinib, was available. The adverse-event network was predominantly star-shaped but contained one closed loop involving first-generation TKIs, osimertinib, and lazertinib. (Fig. 2).

### Overall survival

**Fig. 3:**
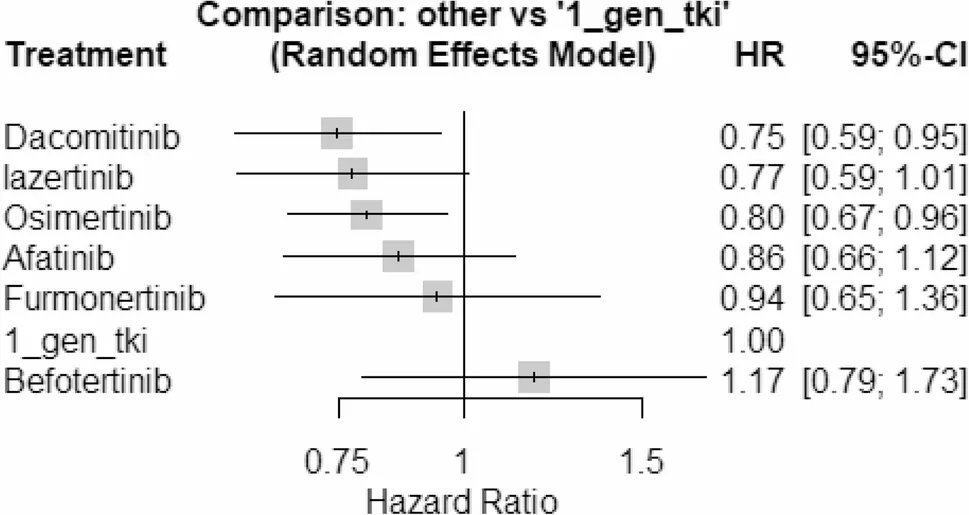
Forest plot of network estimates for overall survival (OS) versus first-generation TKIs. Hazard ratios < 1 favor the intervention over first-generation TKIs

**Fig. 4:**
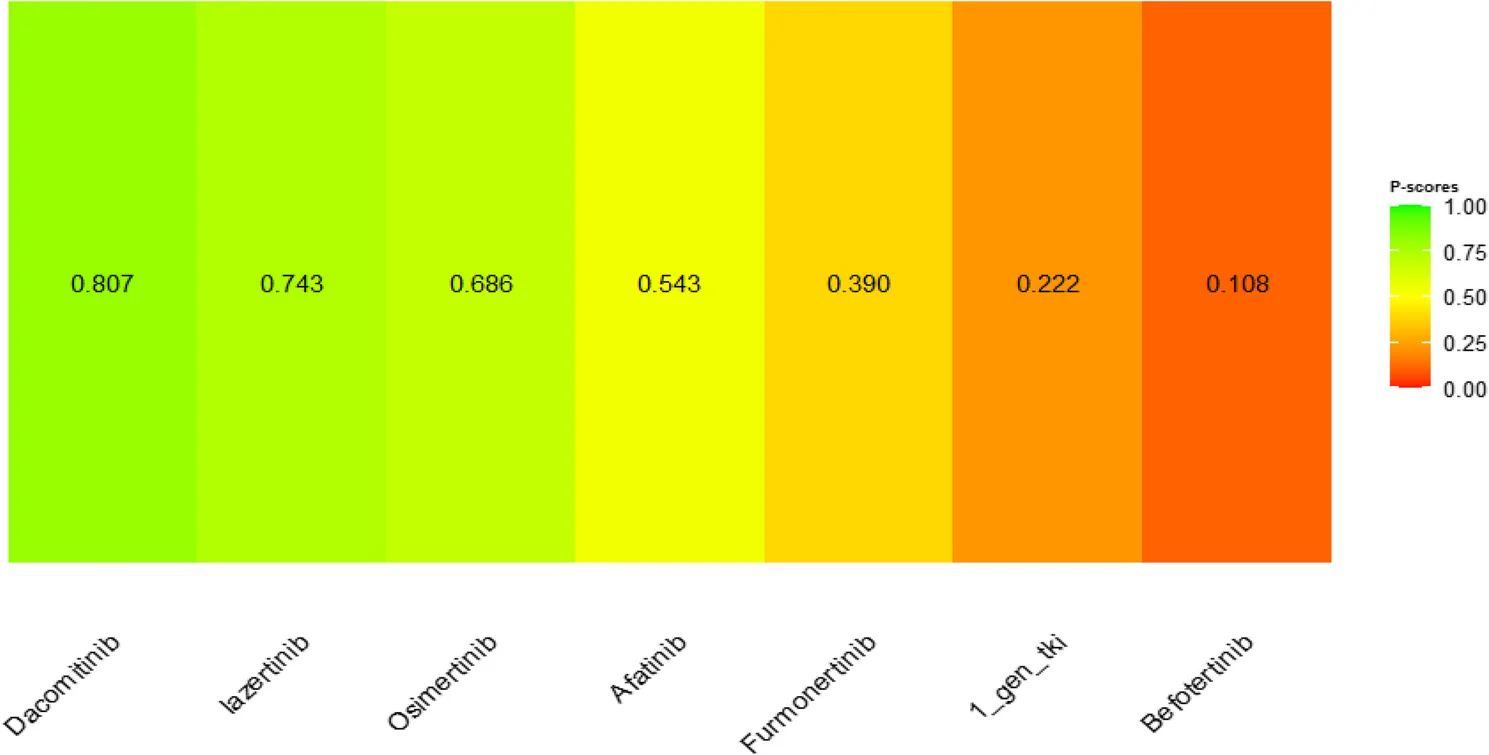
Treatment ranking based on P-scores for overall survival (OS): Higher P-scores indicate a higher relative treatment ranking within the network; rankings should be interpreted together with effect estimates and confidence intervals

Dacomitinib (HR 0.75, 95% CI 0.59–0.95) and Osimertinib (HR 0.80, 95% CI 0.67–0.96) significantly improved OS compared to first-generation TKIs (Fig. 3). Lazertinib showed a trend toward benefit but it did not reach statistical significance. Ranking analysis suggested dacomitinib and lazertinib among top treatments which was followed by osimertinib (Fig. 4). The league table analysis supported the overall network findings and provided indirect comparisons across treatments. No statistically significant differences were observed between most agents, including osimertinib, lazertinib, and afatinib indicating comparable efficacy across these treatments. Similarly, comparisons involving furmonertinib and befotertinib did not demonstrate significant differences in overall survival. Overall, the league table findings were consistent with the network estimates and ranking analysis (Supplementary Table S4).

### Progression-free survival

**Fig. 5:**
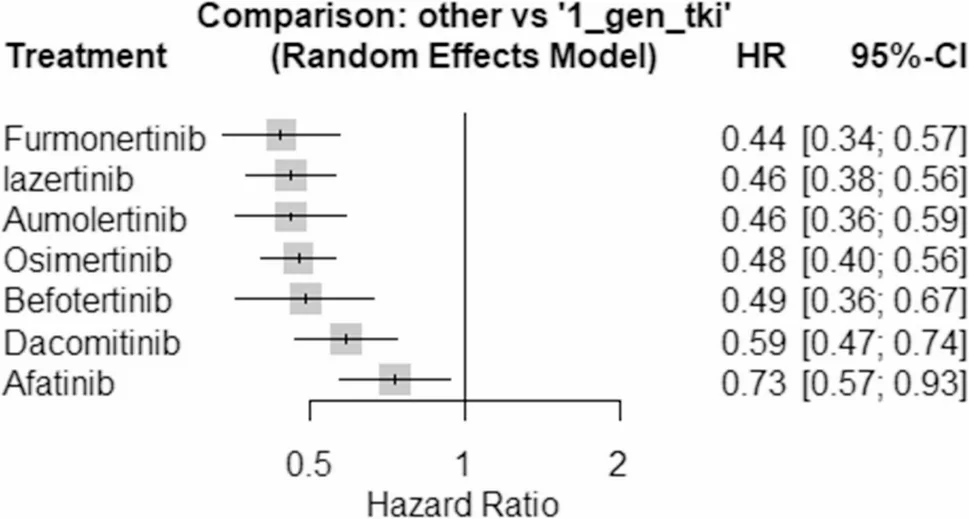
Forest plot of network estimates for progression-free survival (PFS) versus first-generation TKIs. Hazard ratios < 1 favor the intervention over first-generation TKIs

**Fig. 6:**
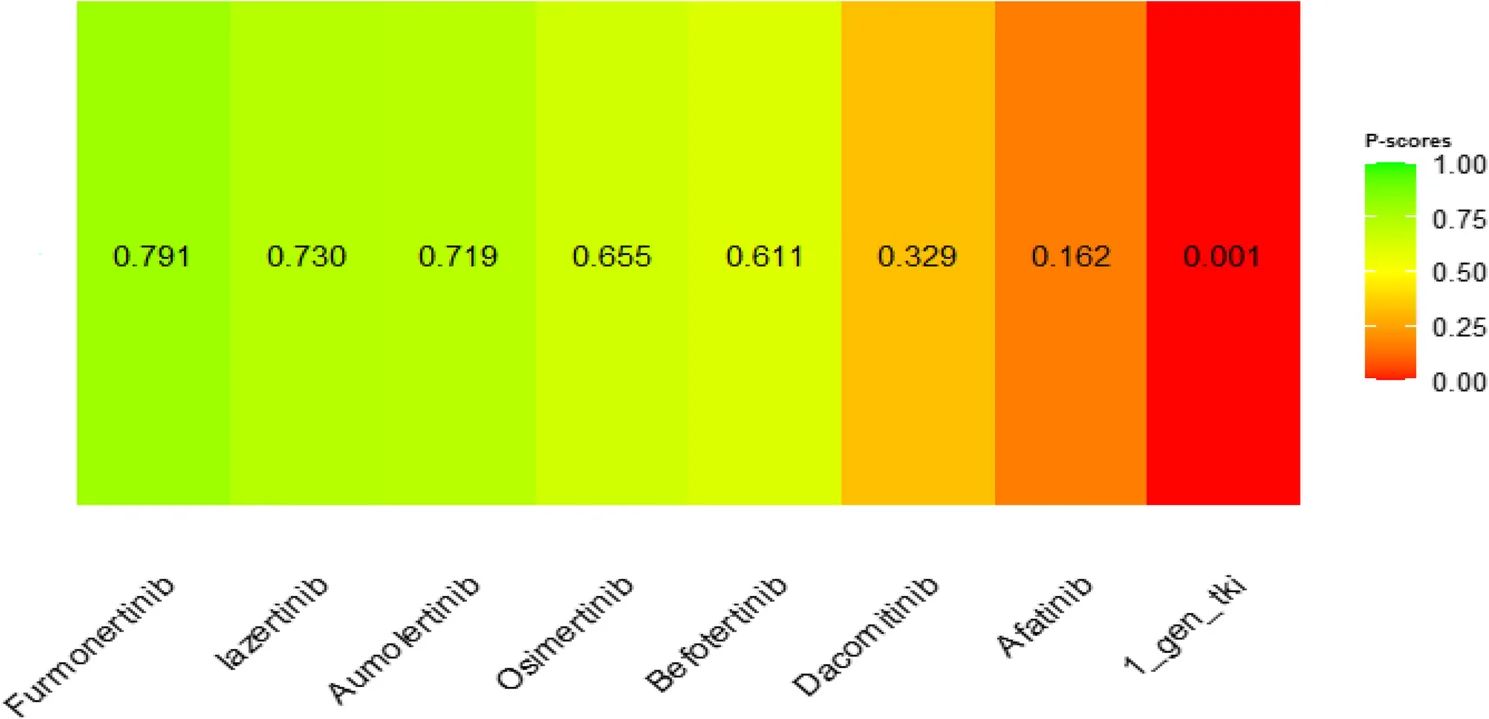
Treatment ranking based on P-score for progression-free survival (PFS)

All newer-generation TKIs significantly improved PFS compared to 1st generation TKIs (Fig. 5). Furmonertinib had the highest P-score for PFS and showed a large benefit versus first-generation TKIs (HR 0.44, 95% CI 0.34–0.57). However, most comparisons among newer-generation TKIs were not statistically significant. Ranking results are shown in Fig. 6. The league table for progression-free survival (Supplementary Table S5) demonstrated consistent benefit across newer agents, including osimertinib, lazertinib, aumolertinib and furmonertinib, with no statistically significant differences observed in most indirect comparisons.

### Grade ≥ 3 adverse events

**Fig. 7:**
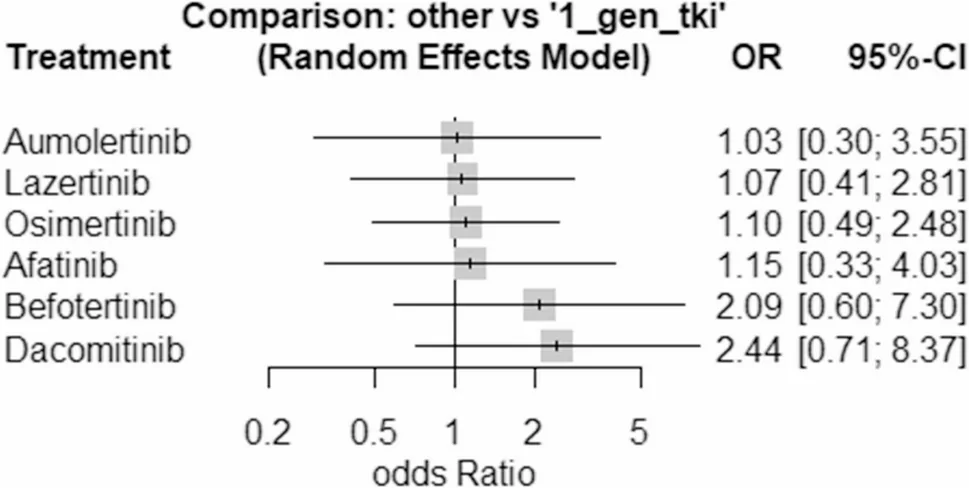
Forest plot of network estimates for grade ≥ 3 adverse events (AE) versus first-generation TKIs. Odds ratios < 1 indicate lower odds of grade ≥ 3 adverse events compared with first-generation TKIs

**Fig. 8:**
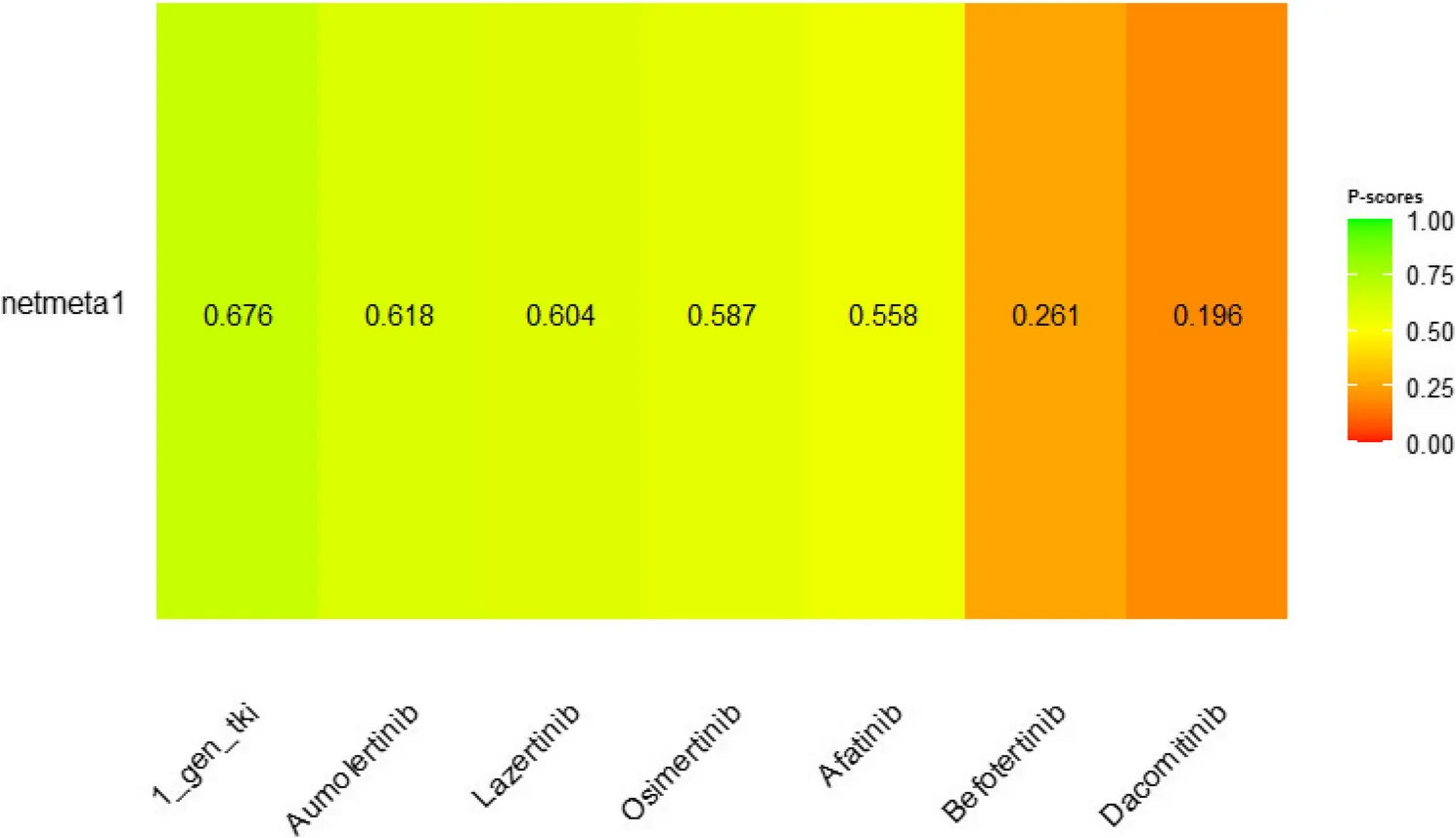
Treatment ranking based on P-scores for grade ≥ 3 adverse events (AE)

In the primary random-effects network meta-analysis, no EGFR-TKI showed a statistically significant difference in grade ≥ 3 adverse events compared with first-generation TKIs. Aumolertinib, lazertinib, osimertinib, and afatinib had broadly comparable safety estimates, whereas befotertinib and dacomitinib showed numerically higher odds of grade ≥ 3 adverse events; however, all confidence intervals crossed the null value (Fig. 7). First-generation TKIs had the highest P-score for safety, followed by aumolertinib, lazertinib, and osimertinib (Fig. 8). The league table analysis demonstrated wide confidence intervals and considerable uncertainty across most treatment comparisons.

### Sensitivity analysis

Sensitivity analyses excluding FLAURA China did not materially alter the OS or PFS estimates, statistical significance, heterogeneity, or treatment rankings; therefore, separate forest plots were not presented. For grade ≥ 3 AEs, exclusion of FLAURA China reduced heterogeneity from I² = 88.1% to 22%. Osimertinib was associated with significantly lower odds than first-generation TKIs, whereas befotertinib and dacomitinib were associated with significantly higher odds (Supplementary Fig S3). In an additional sensitivity analysis excluding the befotertinib-versus-icotinib trial, the direction and statistical significance of the OS, PFS, and grade ≥ 3 adverse-event estimates for the remaining treatments were unchanged, supporting the robustness of the principal conclusions to exclusion of the icotinib-controlled comparison (Supplementary Fig S4). 

### Risk of bias

Risk of bias was assessed using the Cochrane RoB 2 tool. Overall, the included studies were judged to have low risk of bias across most domains, reflecting appropriate randomization procedures, adherence to intended interventions, consistency with protocol-specified analyses, and minimal missing outcome data. The measurement of overall survival was considered low risk in all studies due to its objective nature (Supplementary Table S2).

For adverse events, some concerns were identified in open-label trials as lack of blinding could introduce differential ascertainment. Some concerns were noted for the MARIPOSA lazertinib-versus-osimertinib comparison because it was an exploratory analysis without formal multiplicity adjustment. Overall, the risk of bias across studies was low to some concern, with limited concerns unlikely to substantially affect the validity of the findings (Supplementary Table S3). 

### Consistency and heterogeneity

For overall survival, heterogeneity was low (τ² = 0; I² = 0%, 95% CI 0.0%–89.6%), although the wide confidence interval reflects limited precision. There was no evidence of inconsistency using the design-by-treatment interaction model (between-design Q = 0.12, *p* = 0.734), and within-design heterogeneity was not statistically significant (Q = 0.06, *p* = 0.802).

For progression-free survival, heterogeneity was low (τ² = 0; I² = 0%, 95% CI 0.0%–89.6%), although the wide confidence interval reflects limited precision. There was no evidence of inconsistency using the design-by-treatment interaction model (between-design Q = 0.05, *p* = 0.829), and within-design heterogeneity was not statistically significant (Q = 0.68, *p* = 0.410).

For grade ≥ 3 adverse events, substantial heterogeneity was observed in the primary analysis including FLAURA China (τ² = 0.3584; I² = 88.1%, 95% CI 66.8%–95.7%; total Q = 16.79, *p* = 0.0002). The heterogeneity was primarily attributable to within-design variability (Q = 16.74, *p* < 0.0001), whereas no statistically significant inconsistency was detected between designs (Q = 0.05, *p* = 0.8228). In the sensitivity analysis excluding FLAURA China, heterogeneity decreased substantially (τ² = 0.0093; I² = 22%), and no significant inconsistency was observed (between-design Q = 1.28, *p* = 0.2574) (Supplementary Table S7).

Comparison-adjusted funnel plots for progression-free survival and overall survival did not demonstrate clear asymmetry, suggesting no substantial small-study effects (Supplementary Figures S1 & S2). However, interpretation is limited by the small number of included studies. 

### Certainty of evidence

Certainty of evidence was assessed using a GRADE-based framework adapted for network meta-analysis (Supplementary Table S8). The certainty was rated as high for progression-free survival, moderate for overall survival, and low for grade ≥ 3 adverse events. However, the high certainty rating for PFS primarily reflects comparisons of newer-generation TKIs versus first-generation TKIs; comparisons among newer-generation TKIs were based largely on indirect evidence and should therefore be interpreted with greater caution. The certainty for overall survival was downgraded due to imprecision, as several included trials reported immature survival data. In contrast, progression-free survival estimates were consistent and precise across studies, with no evidence of heterogeneity or inconsistency. For adverse events, the certainty was downgraded due to substantial heterogeneity and variability in adverse event reporting across trials.

## Discussion

This network meta-analysis provides a focused and clinically relevant comparison of EGFR-TKIs in treatment-naïve EGFR-mutant non–small cell lung cancer. While several agents demonstrated superiority over first-generation TKIs in progression-free survival, these benefits did not consistently translate into overall survival advantages. Osimertinib demonstrated consistent efficacy across survival outcomes. In the primary random-effects safety analysis, no EGFR-TKI showed a statistically significant difference compared with first-generation TKIs. However, the sensitivity analysis excluding FLAURA China favoured osimertinib, indicating that the safety findings were sensitive to inclusion of the regional FLAURA analysis. Dacomitinib ranked highly for overall survival but showed numerically higher odds of grade ≥ 3 adverse events. Lazertinib showed a favourable balance between efficacy and tolerability, although most comparisons were not statistically significant. Treatment rankings were considered supplementary to the effect estimates. Although furmonertinib had the highest P-score for PFS, confidence intervals for comparisons among newer-generation TKIs overlapped substantially, and most indirect comparisons were not statistically significant. Therefore, a higher P-score should not be interpreted as definitive evidence of clinical superiority.

Several prior network meta-analyses have evaluated EGFR-TKIs in this setting, often including a larger number of studies. For example, one analysis including 23 randomized trials concluded that osimertinib was the most effective treatment for both progression-free and overall survival [26]. Similarly, another study reported osimertinib and combination strategies among the highest-ranked treatments for survival outcomes. Other analyses also suggested a consistent advantage of third-generation TKIs, particularly osimertinib, in terms of progression-free survival and to a lesser extent overall survival [27, 28].

However, these studies differ substantially from the present analysis in their design and clinical scope. First, the number of included studies in our analysis is smaller compared to previous network meta-analyses. This is primarily due to restrictive and clinically driven inclusion criteria. Unlike prior analyses, we included only head-to-head randomized comparisons between EGFR-TKIs and excluded trials comparing TKIs with chemotherapy or placebo. This focused approach reflects current treatment paradigms, where EGFR-TKIs are the standard first-line therapy and the key clinical question is the optimal TKI selection rather than comparison with chemotherapy.

Another important methodological decision was the grouping of gefitinib, erlotinib, and icotinib into a common first-generation TKI node. These agents share a reversible mechanism of EGFR inhibition and occupy a similar clinical role. Separate modelling was constrained because FLAURA reported gefitinib and erlotinib as a combined comparator, whereas icotinib was represented only in the befotertinib trial. Exclusion of the befotertinib-versus-icotinib trial did not materially alter the findings, suggesting that inclusion of icotinib did not influence the overall network estimates. While some previous network meta-analyses treated these agents separately, such separation may artificially increase network complexity without providing additional clinically meaningful insight [28, 29]. 

### Interpretation of findings

In contrast to earlier NMAs that consistently ranked osimertinib as the most effective therapy, our analysis demonstrates a more nuanced efficacy landscape. While osimertinib remains among the top-performing agents, other third-generation TKIs, including lazertinib and furmonertinib, showed comparable efficacy. This difference likely reflects inclusion of recent trials evaluating newer agents, greater emphasis on direct head-to-head comparisons and reduced influence of indirect comparisons via chemotherapy nodes. A major consideration in this analysis is the inclusion of studies with immature OS data, particularly for newer-generation TKIs. This approach was adopted because many contemporary trials have mature PFS but evolving OS, excluding such studies would limit inclusion of newer agents and bias the results toward older therapies.

Heterogeneity across the network was low for both overall survival and progression-free survival (τ²=0), with no evidence of inconsistency using the design-by-treatment interaction model. However, the wide confidence intervals around I² reflect limited precision due to the relatively small number of included studies and the sparse network structure. Substantial heterogeneity was observed for grade ≥ 3 adverse events in the primary analysis and decreased markedly after exclusion of FLAURA China. FLAURA and FLAURA China used the same study protocol and reported grade ≥ 3 adverse events of any cause; therefore, the heterogeneity between these reports was unlikely to result from differing outcome definitions. The higher event rate in FLAURA China may partly reflect increased local reporting of laboratory- and disease-related adverse events, as well as its smaller sample size, partial participant overlap, and possible regional differences in adverse-event ascertainment. Across the wider network, additional variability may have arisen from differences in adverse-event definitions, open-label versus blinded design, follow-up duration, monitoring intensity, dose-modification practices, and reporting procedures. These factors reduce confidence in indirect safety comparisons and warrant cautious interpretation of the comparative safety estimates.

The network was sparse and largely star-shaped, with first-generation TKIs serving as the principal common comparator. Consequently, most comparisons among newer-generation agents were informed predominantly by indirect evidence. Although no statistically significant inconsistency was detected, the limited number of closed loops and direct head-to-head comparisons reduced the power to identify inconsistency and limited the certainty of these indirect estimates. Apparent differences among newer TKIs should therefore not be interpreted as definitive evidence of superiority.

The certainty of evidence varied across outcomes, being high for progression-free survival, moderate for overall survival, and low for grade ≥ 3 adverse events, reflecting imprecision in OS and substantial heterogeneity in safety outcomes. 

### Strengths

In contrast to previous broader NMAs, this study provides a more focused and contemporary comparison of EGFR-TKIs, reflecting current clinical practice. Our findings suggest that while third-generation TKIs consistently improve progression-free survival, overall survival differences remain less pronounced, and multiple agents demonstrate comparable efficacy. These results highlight the need for individualized treatment selection based on efficacy, safety, and patient-specific factors rather than reliance on a single preferred agent. 

### Limitations

This analysis has several limitations. Despite the multinational nature of several included trials, some racial and ethnic groups were underrepresented, which may limit the generalisability of the findings. The global FLAURA and FLAURA China reports included 19 overlapping participants, introducing limited non-independence and possible slight over-weighting of the osimertinib comparison in the primary network. Both reports were retained because FLAURA China contributed 117 additional participants not included in the global FLAURA population. Sensitivity analyses excluding FLAURA China did not materially alter the OS or PFS findings, although the safety estimates were more sensitive to its exclusion. Overall survival data were immature in several trials, which may affect treatment ranking. The MARIPOSA lazertinib-versus-osimertinib comparison was randomized and double-blind but exploratory and was not powered for definitive superiority or non-inferiority conclusions. Lack of CNS-specific outcomes and subgroup analyses by mutation type within the network framework also limits the scope of interpretation of results. Finally, the limited safety-network structure and heterogeneity in adverse-event definitions and reporting may have affected the comparative safety estimates.

## Conclusion

In this network meta-analysis of treatment-naïve patients with EGFR-mutant advanced NSCLC, all evaluated second- and third-generation EGFR-TKIs improved progression-free survival compared with first-generation TKIs. Osimertinib and dacomitinib were associated with statistically significant overall-survival benefits, whereas estimates for several newer agents remained imprecise because of immature survival data and sparse comparisons. Comparative safety findings were uncertain and sensitive to the inclusion of FLAURA China. No single treatment was consistently superior across all evaluated outcomes. Safety estimates varied across treatments, although comparative differences remained uncertain, underscoring the importance of considering tolerability alongside efficacy in clinical decision-making.

## Supplementary Information

Supplementary Material 1.

Supplementary Material 2.

## Data Availability

No datasets were generated or analysed during the current study.

## Abbreviations

AE: Adverse events
CTCAE: Common Terminology Criteria for Adverse Events
EGFR: Epidermal growth factor receptor
NMA: Network meta-analysis
NSCLC: Non–small cell lung cancer
OS: Overall survival
PFS: Progression-free survival
RCT: Randomized controlled trial
TKI: Tyrosine kinase inhibitor
