# Supplementary material for: Comparative efficacy and safety of first, second, and third-generation EGFR tyrosine kinase inhibitors in treatment-naïve EGFR-mutant advanced non–small cell lung cancer: a systematic review and network meta-analysis of randomized controlled trials
Source: J Egypt Natl Canc Inst. 2026 Sep 14;38:71. doi: 10.1186/s43046-026-00414-2 (PMC13574751; doi:10.1186/s43046-026-00414-2)
Supplement: Supplementary file 1 [file 43046_2026_414_MOESM2_ESM.docx]

**Supplementary Appendix**

**Appendix 1.** Detailed Search Strategy

**Structure:**

**1. PubMed (MEDLINE)**

(("Carcinoma, Non-Small-Cell Lung"[Mesh] OR NSCLC[tiab] OR "non small cell lung cancer"[tiab] OR "non-small-cell lung cancer"[tiab])

AND

("Receptor, Epidermal Growth Factor"[Mesh] OR EGFR[tiab])

AND

(mutation*[tiab] OR "Exon 19"[tiab] OR "Exon 21"[tiab] OR L858R[tiab])

AND

(afatinib[tiab] OR dacomitinib[tiab] OR osimertinib[tiab] OR gefitinib[tiab] OR erlotinib[tiab] OR aumolertinib[tiab] OR furmonertinib[tiab] OR icotinib[tiab] OR befotertinib[tiab] OR "EGFR-TKI"[tiab] OR "tyrosine kinase inhibitor"[tiab])

AND

(randomized controlled trial[pt] OR randomized[tiab] OR randomly[tiab] OR trial[ti]))

**2. Embase**

('non small cell lung cancer'/exp OR 'nsclc':ti,ab OR 'non small cell lung cancer':ti,ab)

AND

('epidermal growth factor receptor'/exp OR egfr:ti,ab)

AND

(mutation*:ti,ab OR 'exon 19':ti,ab OR 'exon 21':ti,ab OR l858r:ti,ab)

AND

(afatinib:ti,ab OR dacomitinib:ti,ab OR osimertinib:ti,ab OR gefitinib:ti,ab OR erlotinib:ti,ab OR aumolertinib:ti,ab OR furmonertinib:ti,ab OR icotinib:ti,ab OR befotertinib:ti,ab OR 'tyrosine kinase inhibitor':ti,ab)

AND

('randomized controlled trial'/exp OR random*:ti,ab OR placebo*:ti,ab)

**3. Scopus**

(TITLE-ABS-KEY ("non small cell lung cancer" OR NSCLC))
AND
(TITLE-ABS-KEY (EGFR AND mutation OR "exon 19" OR "exon 21" OR L858R))
AND
(TITLE-ABS-KEY (afatinib OR dacomitinib OR osimertinib OR gefitinib OR erlotinib OR aumolertinib OR furmonertinib OR icotinib OR befotertinib OR "tyrosine kinase inhibitor"))
AND
(TITLE-ABS-KEY (randomized OR randomised OR trial))

**4. Cochrane Library (CENTRAL)**

("non small cell lung cancer" OR NSCLC)
AND
(EGFR OR "epidermal growth factor receptor")
AND
(mutation OR "exon 19" OR "exon 21" OR L858R)
AND
(afatinib OR dacomitinib OR osimertinib OR gefitinib OR erlotinib OR aumolertinib OR furmonertinib OR icotinib OR befotertinib OR "tyrosine kinase inhibitor")

**Supplementary Table S1.** Excluded Studies

| 1 | Osimertinib after Chemoradiotherapy in Stage III EGFR-Mutated NSCLC. | NCT03521154 | Previously treated / not first-line setting |
| --- | --- | --- | --- |
| 2 | ADAURA: Phase III, Double-blind, Randomized Study of Osimertinib Versus Placebo in EGFR Mutation-positive Early-stage NSCLC After Complete Surgical Resection. | NCT02511106 | Early-stage disease / placebo comparator |
| 3 | Three-Year Safety, Tolerability, and Health-Related Quality of Life Outcomes of Adjuvant Osimertinib in Patients With Resected Stage IB to IIIA EGFR-Mutated NSCLC: Updated Analysis From the Phase 3 ADAURA Trial. | NCT02511106 | Subgroup / secondary analysis |
| 4 | Gefitinib (an EGFR tyrosine kinase inhibitor) plus anlotinib (an multikinase inhibitor) for untreated, EGFR-mutated, advanced non-small cell lung cancer (FL-ALTER): a multicenter phase III trial. | NCT04028778 | Combination therapy (non–head-to-head TKI) |
| 5 | Lazertinib Versus Gefitinib Tyrosine Kinase Inhibitors in Treatment-Naïve Patients With EGFR-Mutated Advanced NSCLC: Analysis of the Asian Subpopulation in LASER301. | NCT04248829 | Subgroup analysis |
| 6 | Central Nervous System Efficacy of Furmonertinib (AST2818) Versus Gefitinib as First-Line Treatment for EGFR-Mutated NSCLC: Results From the FURLONG Study. | NCT03787992 | Subgroup analysis |
| 7 | Lazertinib versus Gefitinib as First-Line Treatment for EGFR-mutated Locally Advanced or Metastatic NSCLC: LASER301 Korean Subset. | NCT04248829 | Subgroup analysis |
| 8 | First-line afatinib vs gefitinib for patients with EGFR mutation-positive NSCLC (LUX-Lung 7): impact of afatinib dose adjustment and analysis of mode of initial progression for patients who continued treatment beyond progression. | NCT01466660 | Subgroup / secondary analysis |
| 9 | The patient's perspective on treatment with dacomitinib: patient-reported outcomes from the Phase III trial ARCHER 1050. | NCT01774721 | Subgroup / secondary analysis |
| 10 | Cost-effectiveness of Osimertinib in the First-Line Treatment of Patients With EGFR-Mutated Advanced Non-Small Cell Lung Cancer. | NCT02296125 | Economic analysis |
| 11 | Adjuvant Erlotinib Versus Placebo in Patients With Stage IB-IIIA Non-Small-Cell Lung Cancer (RADIANT): A Randomized, Double-Blind, Phase III Trial. | NCT00373425 | Placebo comparator |
| 12 | Gefitinib versus placebo in completely resected non-small-cell lung cancer: results of the NCIC CTG BR19 study. | NCT00049543 | Placebo comparator |
| 13 | Final overall survival results from a phase III, randomized, placebo-controlled, parallel-group study of gefitinib versus placebo as maintenance therapy in patients with locally advanced or metastatic non-small-cell lung cancer (INFORM; C-TONG 0804). | NCT00770588 | Maintenance / placebo comparator |
| 14 | Dacomitinib versus erlotinib in patients with advanced-stage, previously treated non-small-cell lung cancer (ARCHER 1009): a randomised, double-blind, phase 3 trial. | NCT01360554 | Previously treated population |
| 15 | A phase III, randomized, open-label study of ASP8273 versus erlotinib or gefitinib in patients with advanced stage IIIB/IV non-small-cell lung cancer. | NCT02588261 | Investigational TKI not relevant to current clinical practice |
| 16 | Randomized phase II study of erlotinib plus tivantinib versus erlotinib plus placebo in previously treated non-small-cell lung cancer. | NCT00777309 | Combination therapy |
| 17 | Phase III Multinational, Randomized, Double-Blind, Placebo-Controlled Study of Tivantinib (ARQ 197) Plus Erlotinib Versus Erlotinib Alone in Previously Treated Patients With Locally Advanced or Metastatic Nonsquamous Non-Small-Cell Lung Cancer. | NCT01244191 | Combination therapy |
| 18 | Rationale and design of MARQUEE: a phase III, randomized, double-blind study of tivantinib plus erlotinib versus placebo plus erlotinib in previously treated patients with locally advanced or metastatic, nonsquamous, non-small-cell lung cancer. | NCT01244191 | Study design |
| 19 | Randomized, double-blind, placebo-controlled, phase II trial of sorafenib and erlotinib or erlotinib alone in previously treated advanced non-small-cell lung cancer. | NCT00600015 | Combination therapy |
| 20 | Icotinib versus gefitinib in previously treated advanced non-small-cell lung cancer (ICOGEN): a randomised, double-blind phase 3 non-inferiority trial. | NCT01040780 | Previously treated population |
| 21 | Randomized phase II study of gefitinib versus erlotinib in patients with advanced non-small cell lung cancer who failed previous chemotherapy. |  | Previously treated population |
| 22 | Erlotinib versus chemotherapy for advanced NSCLC. |  | Non-TKI comparator |
| 23 | Phase II prospective study of the efficacy of gefitinib for the treatment of stage III/IV non-small cell lung cancer with EGFR mutations, irrespective of previous chemotherapy. |  | Non-randomized |
| 24 | A randomized, double-blind, placebo-controlled, phase III trial of erlotinib with or without a c-Met inhibitor tivantinib (ARQ 197) in Asian patients with previously treated stage IIIB/IV nonsquamous NSCLC harboring wild-type EGFR (ATTENTION study). | NCT01377376 | Combination therapy |
| 25 | A Prospective Evaluation of Circulating Tumor Cells and Cell-Free DNA in EGFR-Mutant NSCLC Patients Treated with Erlotinib on a Phase II Trial. | NCT02279004 | Non-comparative |
| 26 | Osimertinib treatment based on plasma T790M monitoring in patients with EGFR-mutant NSCLC (APPLE trial). | NCT02856893 | Non head-to-head TKI design |
| 27 | Randomized Phase II Study of 3 Months or 2 Years of Adjuvant Afatinib in Patients With Surgically Resected Stage I-III EGFR-Mutant NSCLC. | NCT01746251 | Early-stage disease |
| 28 | Randomized Adjuvant Chemotherapy of EGFR-Mutated NSCLC Patients with or without Icotinib Consolidation Therapy. | NCT02430974 | Combination therapy |
| 29 | Erlotinib plus placebo study in NSCLC. |  | Combination therapy |
| 30 | Effectiveness of gefitinib against uncommon EGFR mutations. |  | Non-comparative |
| 31 | Epidermal growth factor receptor-related tumor markers and clinical outcomes with erlotinib in NSCLC: TRUST study. |  | Observational |
| 32 | Dacomitinib compared with placebo in pretreated patients with advanced NSCLC (NCIC CTG BR.26). | NCT01000025 | Placebo comparator |
| 33 | A phase III randomised controlled trial of erlotinib vs gefitinib in advanced NSCLC with EGFR mutations. | NCT01024413 | Mixed/unclear treatment setting |
| 34 | Low-dose gefitinib treatment for advanced NSCLC. |  | Non-randomized |
| 35 | Gefitinib versus Erlotinib as first-line treatment for EGFR mutation-positive NSCLC. |  | Non-randomized |
| 36 | Central Nervous System Outcomes of Lazertinib Versus Gefitinib in EGFR-Mutated Advanced NSCLC. | NCT04248829 | Subgroup analysis |
| 37 | AENEAS subgroup analysis. | NCT03849768 | Subgroup analysis |
| 38 | Phase II EGFR mutation study. |  | Non-randomized |
| 39 | Erlotinib combination study. |  | Combination therapy |
| 40 | EGFR TKI after chemotherapy study. |  | Previously treated population |

**Supplementary Table S2:** RoB 2 Table – Overall Survival/Progression free survival

| **Study (Unique ID)** | **Experimental** | **Comparator** | **D1** | **D2** | **D3** | **D4** | **D5** | **Overall** |
| --- | --- | --- | --- | --- | --- | --- | --- | --- |
| FLAURA | Osimertinib | 1st gen TKI | Low risk | Low risk | Low risk | Low risk | Low risk | Low risk |
| ARCHER 1050 | Dacomitinib | Gefitinib | Low risk | Low risk | Low risk | Low risk | Some concerns | Low risk |
| FLAURA China | Osimertinib | 1st gen TKI | Low risk | Low risk | Low risk | Low risk | Low risk | Low risk |
| FURLONG | Furmonertinib | Gefitinib | Low risk | Low risk | Low risk | Low risk | Low risk | Low risk |
| LUX-Lung 7 | Afatinib | Gefitinib | Low risk | Low risk | Low risk | Low risk | Low risk | Low risk |
| MARIPOSA | Lazertinib | Osimertinib | Low risk | Low risk | Low risk | Low risk | Some concerns | Low risk |
| LASER301 | Lazertinib | Gefitinib | Low risk | Low risk | Low risk | Low risk | Low risk | Low risk |
| Befotertinib (Lu S et al., 2023) | Befotertinib | Icotinib | Low risk | Low risk | Low risk | Low risk | Low risk | Low risk |

**Supplementary Table S3:** RoB 2 Table – Grade ≥3 adverse events.

| **Study (Unique ID)** | **Experimental** | **Comparator** | **D1** | **D2** | **D3** | **D4** | **D5** | **Overall** |
| --- | --- | --- | --- | --- | --- | --- | --- | --- |
| Soria JC (FLAURA) | Osimertinib | 1st gen TKI | Low risk | Low risk | Low risk | Low risk | Low risk | Low risk |
| Cheng Y (FLAURA China) | Osimertinib | 1st gen TKI | Low risk | Low risk | Low risk | Low risk | Low risk | Low risk |
| Park K (LUX-Lung 7) | Afatinib | Gefitinib | Low risk | Low risk | Low risk | Some concerns | Low risk | Some concerns |
| AENEAS | Aumolertinib | Gefitinib | Low risk | Low risk | Low risk | Low risk | Low risk | Low risk |
| ARCHER 1050 | Dacomitinib | Gefitinib | Low risk | Low risk | Low risk | Some concerns | Low risk | Some concerns |
| MARIPOSA | Lazertinib | Osimertinib | Low risk | Low risk | Low risk | Low risk | Some concerns | Some concerns |
| Befotertinib (Lu S et al., 2023) | Befotertinib | Icotinib | Low risk | Low risk | Low risk | Low risk | Some concerns | Some concerns |
| LASER301 | Lazertinib | Gefitinib | Low risk | Low risk | Low risk | Low risk | Low risk | Low risk |

D1: Randomisation process
D2: Deviations from intended interventions
D3: Missing outcome data
D4: Measurement of the outcome
D5: Selection of the reported result

**Supplementary table S4:** League table- Overall Survival

|  | **Dacomitinib** | **Lazertinib** | **Osimertinib** | **Afatinib** | **Furmonertinib** | **1st gen TKI** |
| --- | --- | --- | --- | --- | --- | --- |
| **Dacomitinib** | — | — | — | — | — | — |
| **Lazertinib** | 0.97 (0.68–1.38) | — | — | — | — | — |
| **Osimertinib** | 0.93 (0.69–1.26) | 0.97 (0.75–1.25) | — | — | — | — |
| **Afatinib** | 0.87 (0.61–1.24) | 0.90 (0.62–1.31) | 0.93 (0.68–1.28) | — | — | — |
| **Furmonertinib** | 0.80 (0.51–1.23) | 0.82 (0.52–1.30) | 0.85 (0.56–1.29) | 0.91 (0.58–1.44) | — | — |
| **1st gen TKI** | 0.75 (0.59–0.95) | 0.77 (0.59–1.01) | 0.80 (0.67–0.96) | 0.86 (0.66–1.12) | 0.94 (0.65–1.36) | — |
| **Befotertinib** | 0.64 (0.40–1.01) | 0.66 (0.41–1.06) | 0.68 (0.44–1.06) | 0.74 (0.46–1.18) | 0.80 (0.47–1.38) | 0.85 (0.58–1.27) |

Note- Values are presented as hazard ratios (HRs) comparing column treatments to row treatments. HR < 1 favors the column treatment.

**Supplementary Table S5:** League table- Progression free survival

|  | **Furmonertinib** | **Lazertinib** | **Aumolertinib** | **Osimertinib** | **Befotertinib** | **Dacomitinib** | **Afatinib** |
| --- | --- | --- | --- | --- | --- | --- | --- |
| **Furmonertinib** | — | — | — | — | — | — | — |
| **Lazertinib** | 0.96 (0.69–1.33) | — | — | — | — | — | — |
| **Aumolertinib** | 0.96 (0.67–1.37) | 1.00 (0.73–1.37) | — | — | — | — | — |
| **Osimertinib** | 0.93 (0.68–1.26) | 0.97 (0.81–1.16) | 0.97 (0.72–1.30) | — | — | — | — |
| **Befotertinib** | 0.90 (0.60–1.34) | 0.94 (0.65–1.36) | 0.94 (0.63–1.39) | 0.97 (0.68–1.38) | — | — | — |
| **Dacomitinib** | 0.75 (0.53–1.05) | 0.78 (0.57–1.06) | 0.78 (0.56–1.09) | 0.81 (0.61–1.07) | 0.83 (0.57–1.22) | — | — |
| **Afatinib** | 0.60 (0.42–0.86) | 0.63 (0.46–0.87) | 0.63 (0.44–0.89) | 0.65 (0.48–0.88) | 0.67 (0.45–1.00) | 0.81 (0.58–1.13) | — |
| **1st gen TKI** | 0.44 (0.34–0.57) | 0.46 (0.38–0.56) | 0.46 (0.36–0.59) | 0.48 (0.40–0.56) | 0.49 (0.36–0.67) | 0.59 (0.47–0.74) | 0.73 (0.57–0.93) |

Note- Values are presented as hazard ratios (HRs) comparing column treatments to row treatments. HR < 1 favors the column treatment.

**Supplementary Table S6:** League table- Grade 3 and above AE

| **Row treatment** | **1st-generation TKI** | **Afatinib** | **Aumolertinib** | **Befotertinib** | **Dacomitinib** | **Lazertinib** | **Osimertinib** |
| --- | --- | --- | --- | --- | --- | --- | --- |
| **1st-generation TKI** | — |  |  |  |  |  |  |
| **Afatinib** | 0.87 (0.25–3.04) | — |  |  |  |  |  |
| **Aumolertinib** | 0.97 (0.28–3.35) | 1.12 (0.19–6.51) | — |  |  |  |  |
| **Befotertinib** | 0.48 (0.14–1.67) | 0.55 (0.09–3.23) | 0.49 (0.08–2.86) | — |  |  |  |
| **Dacomitinib** | 0.41 (0.12–1.41) | 0.47 (0.08–2.73) | 0.42 (0.07–2.42) | 0.86 (0.15–4.96) | — |  |  |
| **Lazertinib** | 0.93 (0.36–2.44) | 1.07 (0.22–5.20) | 0.96 (0.20–4.59) | 1.95 (0.40–9.43) | 2.27 (0.48–10.86) | — |  |
| **Osimertinib** | 0.91 (0.40–2.03) | 1.04 (0.23–4.62) | 0.93 (0.21–4.08) | 1.89 (0.43–8.39) | 2.21 (0.51–9.64) | 0.97 (0.37–2.53) | — |

Odds ratios (ORs) compare column treatments to row treatments. OR < 1 favors the column treatment (fewer adverse events).

**Supplementary Table S7:** Assessment of consistency using the design-by-treatment interaction model for

**Overall Survival (OS)**

Quantifying heterogeneity / inconsistency:

tau^2 = 0; tau = 0; I^2 = 0% [0.0%; 89.6%]

Tests of heterogeneity (within designs) and inconsistency (between designs):

Q	d.f. p-value

Total	0.18	2	0.9148

Within designs	0.06	1	0.8018

Between designs 0.12	1	0.7344

**Progression free survival (PFS)**

Q statistics to assess homogeneity / consistency (Global assessment)

Q df p-value

Total	0.72 2 0.6960

Within designs 0.68 1 0.4101

Between designs 0.05 1 0.8294

*Design-specific decomposition* of within-designs Q statistic

Design	Q df p-value

1_gen_tki:Osimertinib 0.68 1 0.4101

Between-designs Q statistic after detaching of single designs

Detached design	Q df p-value

1_gen_tki:lazertinib 0.00 0	--

1_gen_tki:Osimertinib 0.00 0	--

lazertinib:Osimertinib 0.00 0	--

Q statistic to assess consistency under the assumption of

a full **design-by-treatment** interaction random effects model

Q df p-value tau.within tau2.within

Between designs 0.05 1 0.8294	0	0

Note- Q statistics were derived using the design-by-treatment interaction model. Total Q reflects overall variability, within-design Q reflects heterogeneity, and between-design Q assesses inconsistency. τ² and I² represent the magnitude of heterogeneity. A p-value >0.05 indicates no statistically significant heterogeneity or inconsistency.

**Adverse Event (AE)**

Quantifying heterogeneity / inconsistency:

tau^2 = 0.3584; tau = 0.5986; I^2 = 88.1% [66.8%; 95.7%]

Tests of heterogeneity (within designs) and inconsistency (between designs):

Q	d.f. p-value

Total	16.79	2	0.0002

Within designs 16.74	1 < 0.0001

Between designs 0.05	1	0.8228

Without FLAURA China

Quantifying heterogeneity / inconsistency:

tau^2 = 0.0093; tau = 0.0965; I^2 = 22%

Tests of heterogeneity (within designs) and inconsistency (between designs):

Q	d.f. p-value

Total	1.28	1 0.2574

Within designs	0.00	0	--

Between designs 1.28	1 0.2574

# **Supplementary Table S8: Certainty of Evidence (GRADE Framework) for Network Meta-Analysis Outcomes**

| **Outcome** | **Risk of Bias** | **Inconsistency** | **Indirectness** | **Imprecision** | **Publication Bias** | **Overall Certainty** |
| --- | --- | --- | --- | --- | --- | --- |
| Overall Survival (OS) | Low | Not serious | Not serious | Serious | Undetected | **Moderate** |
| Progression-Free Survival (PFS) | Low | Not serious | Not serious | Not serious | Undetected | **High** |
| Grade ≥3 Adverse Events | Some concerns | Serious | Not serious | Serious | Undetected | **Low** |

Overall Survival (OS): The certainty of evidence for overall survival was rated as moderate. Most included studies were phase III randomized controlled trials with low risk of bias. There was no evidence of inconsistency across the network (Q = 0.12, p = 0.73), and indirectness was minimal as all studies evaluated first-line EGFR-mutated advanced NSCLC populations. However, the certainty was downgraded for imprecision, as several trials reported immature overall survival data, with limited event numbers and wide confidence intervals in some comparisons.

Progression-Free Survival (PFS): The certainty of evidence for progression-free survival was rated as high. The included studies were well-conducted randomized trials with low risk of bias. There was no significant heterogeneity or inconsistency (between-design Q = 0.05, p = 0.829), and effect estimates were precise with narrow confidence intervals. Direct and indirect evidence were consistent, supporting robust network estimates.

Grade ≥3 Adverse Events: The certainty of evidence for grade ≥3 adverse events was rated as low. Although most studies were randomized trials, some had open-label designs, introducing potential bias in adverse event reporting. Additionally, there was substantial heterogeneity (I² = 88.1%), likely reflecting differences in adverse event definitions (treatment-related vs all-cause) and reporting practices across trials. The certainty was further downgraded for imprecision due to variability in event rates and sample sizes across comparisons.

**Supplementary Fig S1: Funnel plot: Overall survival**

**Supplementary Fig S2: Funnel plot: PFS**

**Supplementary Fig S3:** Forest plot for grade ≥3 adverse events excluding FLAURA China.

**Supplementary Fig S4.** Sensitivity analyses excluding the befotertinib-versus-icotinib trial: (A) overall survival, (B) progression-free survival, and (C) grade ≥3 adverse events. Estimates are from random-effects network meta-analyses versus first-generation EGFR-TKIs. Hazard ratios below 1 favour the intervention for survival outcomes; odds ratios below 1 indicate fewer grade ≥3 adverse events.
